# Effectiveness of acupuncture and related therapies for palliative care of cancer: overview of systematic reviews

**DOI:** 10.1038/srep16776

**Published:** 2015-11-26

**Authors:** Xinyin Wu, Vincent CH Chung, Edwin P Hui, Eric TC Ziea, Bacon FL Ng, Robin ST Ho, Kelvin KF Tsoi, Samuel YS Wong, Justin CY Wu

**Affiliations:** 1 Hong Kong Institute of Integrative Medicine, The Chinese University of Hong Kong, Hong Kong.; 2 Jockey Club School of Public Health and Primary Care, The Chinese University of Hong Kong, Hong Kong.; 3 Comprehensive Cancer Trials Unit, The Chinese University of Hong Kong, Hong Kong.; 4 Chinese Medicine Department, Hong Kong Hospital Authority, Hong Kong.; 5 Big Data Decision Analytics Research Centre, The Chinese University of Hong Kong, Hong Kong.; 6 Department of Medicine & Therapeutics, The Chinese University of Hong Kong, Hong Kong.

## Abstract

Acupuncture and related therapies such as moxibustion and transcutaneous electrical nerve stimulation are often used to manage cancer-related symptoms, but their effectiveness and safety are controversial. We conducted this overview to summarise the evidence on acupuncture for palliative care of cancer. Our systematic review synthesised the results from clinical trials of patients with any type of cancer. The methodological quality of the 23 systematic reviews in this overview, assessed using the Methodological Quality of Systematic Reviews Instrument, was found to be satisfactory. There is evidence for the therapeutic effects of acupuncture for the management of cancer-related fatigue, chemotherapy-induced nausea and vomiting and leucopenia in patients with cancer. There is conflicting evidence regarding the treatment of cancer-related pain, hot flashes and hiccups, and improving patients’ quality of life. The available evidence is currently insufficient to support or refute the potential of acupuncture and related therapies in the management of xerostomia, dyspnea and lymphedema and in the improvement of psychological well-being. No serious adverse effects were reported in any study. Because acupuncture appears to be relatively safe, it could be considered as a complementary form of palliative care for cancer, especially for clinical problems for which conventional care options are limited.

Cancer is a major cause of disease burden worldwide. According to estimates from the International Agency for Research of Cancer^1^, the global adult population in 2012 included 14.1 million new cancer cases, 32.6 million existing cancer patients who had received a diagnosis within the previous 5 years and 8.2 million cancer deaths, accounting for 14.7% of all deaths. The incidence of cancer continues to increase. It is predicted that in 2035, approximately 24.0 million new cancer cases will be diagnosed and 14.6 million deaths will be attributable to cancer. This increasing cancer incidence and the continual improvement in cancer treatment will lead to an increase in the number of patients living with cancer. This will mandate progress in palliative care strategies for the control of symptoms related to cancer itself, as well as symptoms induced by cancer therapies.

According to the World Health Organisation^2^, palliative care is an approach that aims to improve the quality of life (QoL) of patients with life-threatening illnesses by relieving pain, distressing symptoms or other side effects related to treatment. Unlike the traditional concept of confining palliative care to the last 6 months of life, the current model recommended by the US National Comprehensive Cancer Network extends palliative care across the entire disease trajectory. Based on this concept, cancer palliative care focuses not only on the treatment of cancer-related symptoms such as pain, fatigue and insomnia, but also on relief of the side effects of chemotherapy or radiotherapy such as nausea, vomiting, leukopenia and xerostomia^3^. This shift in the treatment model is partly related to the failure of aggressive care such as prolonged hospitalisation or intensive care unit admission to increase the life expectancy or improve the QoL of patients with incurable cancer^4^.

Acupuncture is one of the major treatment methods in traditional Chinese medicine. Related therapies such as moxibustion and transcutaneous electrical nerve stimulation (TENS) are also often used separately or in combination with acupuncture. These therapies are widely used for supportive and palliative care of patients with cancer^5^. The existing systematic reviews (SRs) have summarised the evidence on the use of acupuncture and related therapies for the management of various cancer-related symptoms, including nausea and vomiting^6^,^7^, cancer-related pain (CRP)^7^,^8^ and fatigue^7^,^9^. These SRs have drawn contradictory conclusions. For example, Posadzki and colleagues^9^ reviewed seven randomised controlled trials (RCTs) that assessed the effectiveness of acupuncture and related therapies in the treatment of cancer-related fatigue (CRF) and concluded that the risk of bias (RoB) amongst the included RCTs was too high for any reliable conclusions to be drawn. In contrast, another SR^7^ suggested that acupuncture is useful for the reduction of fatigue on the basis of one well-designed RCT. The disagreement amongst these four SRs could be attributed to differences in their inclusion and exclusion criteria, search strategies, databases and search periods.

The contradictory results of these individual SRs make it difficult to draw conclusions on the potential effectiveness of acupuncture and related therapies. An overview of the existing SRs is needed to provide an update on all synthesised clinical evidence on acupuncture and related therapies for palliative cancer care^10^. Although such an overview^11^ has already been conducted, its trustworthiness is limited for the following reasons: first, the search dates were limited to 2000 to 2011; hence, SRs published outside this time frame, including those published or updated since^8^,^9^,^12^,^13^,^14^, were not included. Second, the search strategy of the previous overview did not include Chinese-language databases, which may have led to the omission of clinical evidence^15^. Third, the methodological quality of the included SRs was not appraised with a validated instrument^16^, which limits the interpretation of their overall trustworthiness.

To overcome these limitations, we conducted an up-to-date overview of SRs to evaluate the methodological quality of SRs and meta-analyses of acupuncture for management of symptoms for palliative care of cancer and to describe the clinical evidence reported in these SRs and meta-analyses.

## Results

### SR search and screening results

**Figure 1:**
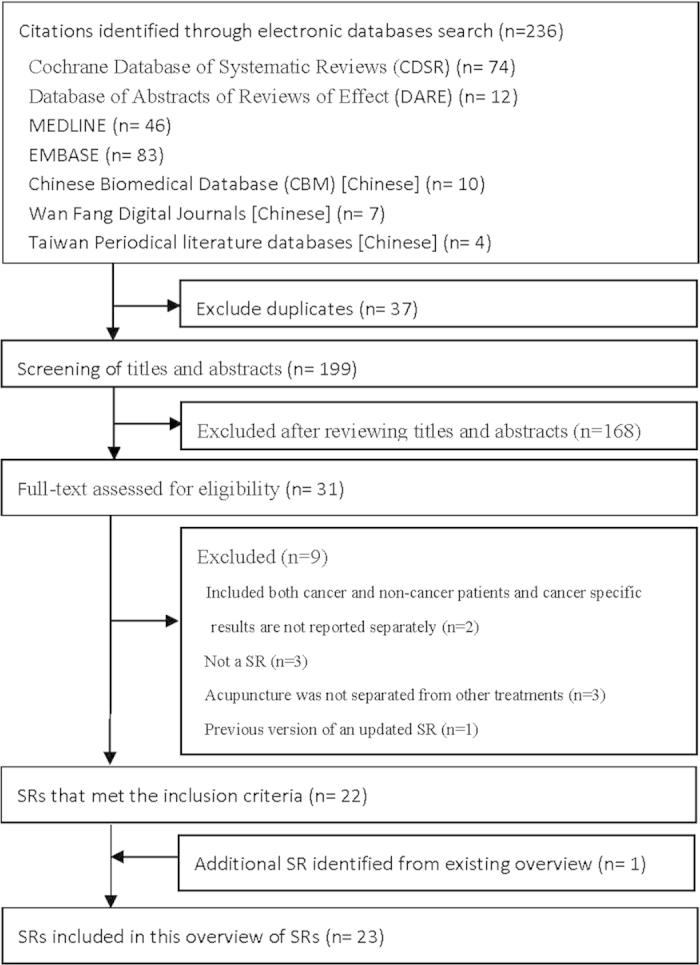
Flowchart of literature selection on systematic reviews of acupuncture for cancer palliative care Keys: SR, systematic review.

The database search strategies yielded 236 records, and 37 duplicates were identified and excluded. We excluded 168 citations after screening the titles and abstracts; thus, the full texts of the remaining 31 citations were retrieved for further assessment. Nine publications were excluded for the following reasons: two included patients with and without cancer and did not report the cancer-specific results separately, three were not SRs, three did not report the results of acupuncture and related therapies separately from those of other treatments and one was an early version of an updated SR. After this process, 22 SRs met the inclusion criteria. We further identified one additional SR by checking the reference list of a previous overview^11^. The other nine SRs included in that overview^11^ had already been identified by the literature search. Thus, a total of 23 SRs were included in this overview. Details of the literature search and selection can be found in Fig. 1.

### Characteristics of included SRs

The 23 SRs were published between 2005 and 2014, and 14 (60.9%) of them were published after 2012. This overview includes 14 new SRs that were not included in the previous overview. Of the 22 SRs that provided a cut-off date on their literature search, 12 (55.0%) conducted their literature search after 2011 (i.e., after the publication of the previous overview^11^).

Overall, these SRs reported the results from 248 primary studies (median, 7) and 17,392 patients (median, 548). Three SRs (13.0%) were published in Chinese, and the rest were written in English. Three (13.0%) were Cochrane SRs. Thirteen (56.5%) SRs included only RCTs, and the rest included multiple study designs, including both clinical trials and observational studies. Seventeen (73.9%) of the SRs covered various types of cancer. Three SRs focused on patients with breast cancer^6^,^7^,^17^, and three other SRs summarised only evidence on patients with prostate cancer^18^, head and neck cancer^19^ and lung cancer^20^. Eleven SRs^6^,^7^,^14^,^18^,^20^,^21^,^22^,^23^,^24^,^25^,^26^ included any type of acupuncture or related therapy either with or without needle insertion. Nine SRs^8^,^9^,^12^,^17^,^19^,^27^,^28^,^29^,^30^ included only acupuncture with needle insertion and excluded other forms of related therapy, including TENS, laser acupuncture, acupressure and moxibustion. The remaining three SRs focused only on one particular form of acupuncture or related therapy, including TENS^13^, moxibustion^31^ and acupoint injection^32^.

Sixteen SRs summarised the evidence on a single outcome, including CRP^8^,^13^,^21^,^22^,^26^,^27^, fatigue^9^,^24^,^31^, hot flashes^17^,^18^, chemotherapy-induced nausea and vomiting (CINV)^14^,^20^, hiccups^29^ and irradiation-induced xerostomia^19^. The remaining seven SRs^6^,^7^,^12^,^20^,^28^,^30^,^32^ reported evidence on a wide range of outcomes in the palliative care of patients with cancer. The characteristics of these SRs can be found in Tables 1, 2, 3. Table 1 describes the SRs that included only RCTs on needle acupuncture. Table 2 reports the SRs that included RCTs that focused on acupuncture-related therapies. Table 3 highlights the SRs that included results from various study designs on both needle acupuncture and related therapies. The methodological quality of the included SRs as assessed by the Methodological Quality of Systematic Reviews (AMSTAR) is shown in Table 4 and Appendix 3. The corresponding results from these SRs can be found in Tables 5, 6, 7 and are supplemented with quantitative results reported in Appendix 4.

### Methodological quality of included SRs

All of the SRs conducted a comprehensive literature search (AMSTAR Item 3). Twenty-two (95.7%) assessed the scientific quality of the included studies (AMSTAR Item 7). Only five (21.7%) provided an a priori design to the SRs (AMSTAR Item 1). Although all of the SRs provided references to the included studies, only six (26.1%) provided lists of both included and excluded studies (AMSTAR Item 5). None of the SRs stated any conflict of interest on the included studies as well as on the SR itself (AMSTAR Item 11). Only 13 SRs (56.5%) included conflict of interest of the SR itself (AMSTAR Item 11). Of the 11 SRs that conducted meta-analyses, eight (81.8%) used appropriate statistical methods to combine the study findings (AMSTAR Item 9), but none assessed the likelihood of publication bias (AMSTAR Item 10). The details can be found in Table 4 and Appendix 3.

### Effectiveness of acupuncture and related therapies

#### Cancer-related pain

Ten SRs^6^,^7^,^8^,^12^,^13^,^21^,^22^,^26^,^27^,^32^ synthesised clinical evidence on the use of acupuncture and related therapies in the reduction of CRP, of which only two^22^,^26^ conducted a meta-analysis (Table 7, Appendix 4). Zheng and colleagues^26^ included five studies (395 patients) that evaluated the effectiveness of wrist-ankle acupuncture on the symptom improvement rate. Their results showed no statistical differences in the symptom improvement rate between wrist-ankle acupuncture plus conventional care and conventional care alone (pooled risk ratio [RR], 1.12; 95% confidence interval [CI], 0.92 to 1.36). Choi and colleagues^22^ summarised evidence from various forms of acupuncture and related therapies, and their synthesised results showed that acupuncture and related therapies plus conventional care had a slightly stronger effect in the reduction of CRP (pooled RR, 1.36; 95% CI, 1.13 to 1.64) than conventional care alone. However, no statistical differences were found in the response rates of acupuncture and related therapies and those of conventional analgesic treatment (pooled RR, 1.12; 95% CI, 0.98 to 1.28). No significant difference was seen in the pain score between acupuncture (including auricular acupuncture and electro-acupuncture) and sham acupuncture (pooled standard mean difference [SMD], –0.41; 95% CI, –1.39 to 0.49).

Meta-analysis was not conducted in the other SRs^6^,^7^,^8^,^13^,^21^,^27^,^32^ (Tables 5, 6, 7 and Appendix 4). Three SRs^8^,^21^,^27^ (Tables 5 and 7 and Appendix 4) identified one RCT with a low RoB and mentioned that auricular acupuncture provided statistically significant relief on CRP when compared with sham acupuncture.

#### Cancer-related fatigue

Five SRs^7^,^9^,^24^,^28^,^31^ summarised evidence on the effectiveness of acupuncture and related therapies in the treatment of CRF, and two conducted meta-analyses (Tables 5, 6, 7 and Appendix 4). Zeng and colleagues^28^ assessed the effectiveness of acupuncture for CRF in various types of cancer patients (Table 5 and Appendix 4). Four meta-analyses were conducted for different comparisons, and all results showed a reduction of CRF with acupuncture. However, only one meta-analysis showed that acupuncture plus education could significantly reduce CRF in cancer patients when compared to conventional care (two RCTs; pooled SMD, –2.12; 95% CI, –3.21 to –1.03). One of the pooled RCTs provided information on the net benefit of acupuncture. In this trial, acupuncture plus conventional care was compared with conventional care alone; the mean difference was –2.52 (95% CI, –2.85 to –2.19). However, the validity of these results was compromised because the two RCTs did not implement allocation concealment and blinding.

Another SR^31^ investigated the effects of moxibustion on CRF (Table 6 and Appendix 4). The pooled results demonstrated that moxibustion plus conventional care could significantly improve CRF over conventional care alone (pooled RR, 1.73; 95% CI, 1.29 to 2.32), but it is uncertain whether the outcomes were assessed with validated scales. Readers should note that all four trials included in this meta-analysis had poor reporting quality, so their RoB was unclear.

#### Hot flashes

Five SRs^6^,^7^,^17^,^18^,^25^ summarised evidence on acupuncture and related therapies for the treatment of hot flashes in patients with breast cancer^6^,^7^,^17^, prostate cancer^18^, or both^25^ (Tables 5 and 7 and Appendix 4). Only one study^17^ conducted a meta-analysis to compare acupuncture with sham acupuncture (Table 5 and Appendix 4). The results favoured acupuncture in the reduction of the frequency of hot flashes during treatment (pooled weighted mean difference [WMD], 1.91; 95% CI, 0.10 to 3.71). However, this difference was attenuated after treatment (pooled WMD, 3.09; 95% CI, –0.04 to 6.23). In contrast, one SR^28^ investigated the long-term effects of acupuncture (3 to 21 months of follow-up) and concluded that its benefits could be sustained for at least 3 months in women with breast cancer and in men with prostate cancer (Table 5 and Appendix 4).

### Nausea and vomiting

Six SRs^6^,^7^,^14^,^20^,^30^,^32^ synthesised evidence on the effectiveness of acupuncture and related therapies on CINV (Tables 5, 6, 7 and Appendix 4). Four^14^,^20^,^30^,^32^ SRs conducted meta-analyses. Two^20^,^32^ reported that the results of acupuncture and related therapies for CINV were better than those of the control group (no details on the control intervention were provided). Another SR^14^ found that acupuncture could reduce the proportion of patients who experienced acute vomiting but did not reduce the mean number of delayed vomiting episodes or the severity of acute or delayed nausea. The remaining SR^30^ suggested that acupuncture and conventional medications had similar effects in the reduction of CINV, but this conclusion was based on a single RCT with an unclear RoB.

#### Quality of life

Four SRs^12^,^20^,^28^,^32^ synthesised evidence on the effectiveness of acupuncture and related therapies for improving cancer patients’ QoL (Tables 5, 6 and Appendix 4). Two SRs^20^,^28^ conducted meta-analyses; one^20^ reported that acupuncture improves QoL in patients with lung cancer when compared to conventional care alone (pooled SMD, 0.47; 95% CI, 0.04 to 0.90), but the other^28^ found no favourable effects of acupuncture when compared to sham acupuncture (pooled SMD, 0.99; 95% CI, –0.70 to 2.68). The results of the two SRs^12^,^32^ that narratively summarised the results of the included RCTs described the favourable effects of acupuncture and related therapies in improving QoL when compared with conventional care or chemotherapy alone. It should be highlighted that only one SR^12^ reported a low RoB for all included trials, and the other three SRs^12^,^20^,^32^ did not provide details on the RoB of the included studies.

#### Leucopenia

Two SRs^6^,^23^ summarised the effectiveness of acupuncture and related therapies in the reversal of cancer treatment–induced leucopenia (Table 7 and Appendix 4). One SR^23^ focused on chemotherapy-induced leucopenia, and the results of the meta-analysis demonstrated that acupuncture promoted an increase in the white blood cell count (pooled WMD, 1.22; 95% CI, 0.64 to 1.81). The other SR^6^ reported a positive effect on radiotherapy-induced leucopenia, but this conclusion was supported by only one small case series with seven patients.

### Lymphedema

Two SRs^6^,^7^ reported the results from one case series study, which concluded that acupuncture was effective in reducing lymphedema related to breast cancer. It was mentioned that the patients experienced significant improvement in their range of movements for shoulder flexion and abduction of the affected limb. Attention should be paid to the lack of a control group and the small sample size (29 patients) in this study (Table 7 and Appendix 4).

### Hiccups

Choi and colleagues^29^ reviewed the effectiveness of needle or electro-acupuncture in the relief of hiccups in cancer patients (Table 5 and Appendix 4). Their meta-analysis showed that acupuncture can significantly reduce hiccups (pooled RR, 1.87; 95% CI, 1.26 to 2.78) when compared with conventional care alone. It should be noted that all three included studies had unclear RoB and that the reliability of the outcome measurement approaches were doubtful.

### Xerostomia

An SR^19^ assessed the effectiveness of needle or electro-acupuncture for the treatment of irradiation-induced xerostomia in patients with head and neck cancer (Table 7 and Appendix 4). All three trials reported a significant reduction in xerostomia compared to baseline salivary flow rates after acupuncture or sham acupuncture. There was no significant difference between the acupuncture and sham acupuncture groups.

### Dyspnea

Dos Santos and colleagues^7^ identified one RCT with a low RoB that evaluated the effects of acupuncture in the treatment of dyspnea in patients with breast cancer (Table 7 and Appendix 4). Patients in both the acupuncture and sham acupuncture groups showed statistically significant improvement in their dyspnea scores immediately after treatment. Although the improvement was slightly greater in the acupuncture group, it was not significantly different from the sham acupuncture group.

### Psychological well-being

The effect of acupuncture on psychological well-being was discussed in two SRs^7^,^28^; however, neither found any significant beneficial effect of acupuncture when compared to a control group.

### Adverse effects of acupuncture and related therapies

Of the 23 SRs, six^7^,^8^,^20^,^21^,^24^,^25^ did not describe any information on adverse events, two^23^,^29^ reported that the included studies did not mention any adverse events and two^22,30^ mentioned that all of the included studies reported no adverse events. The remaining 13 SRs^6^,^9^,^12^,^13^,^14^,^17^,^18^,^19^,^26^,^27^,^28^,^31^,^32^ reported adverse events that were attributable to acupuncture and related therapies. A wide range of minor adverse events was reported, which including bleeding at the acupuncture site, skin irritation, discomfort, transient rash, electrical shock sensation and tingling. There were no reports of serious AE that required medical management.

## Discussion

This updated overview of 23 SRs summarised the clinical evidence on the effectiveness of acupuncture in palliative care for cancer from 248 primary studies that included 17,392 participants. The effects of acupuncture on 12 outcomes that are highly relevant to palliative care of cancer, including CRP, CRF, hot flashes, vomiting and nausea, QoL, leucopenia, lymphedema, hiccups, xerostomia, dyspnea, psychological well-being and adverse events, were synthesised. Compared to the previous overview^11^, we identified and reported results from 14 additional, updated SRs. The clinical evidence from this overview suggests that acupuncture and related therapies are likely to be effective in improving CRF, CINV and leucopenia. In the context of current best practice, acupuncture and related therapies may be considered as additional treatments to the guideline-recommended treatment with glucocorticoids, 5-HT_3_ antagonists and/or NK_1_R antagonists in patients with moderate or high risk of emesis^33^. For leucopenia, antibacterial and antifungal prophylaxis are currently not recommended for patients with solid tumors who are undergoing conventional chemotherapy^34^. Acupuncture and related therapies may serve as an alternative prophylaxis, with a therapeutic goal of reducing the duration of neutropenia (absolute neutrophil count of less than 1500 cells/μL) to less than 7 days. Finally, acupuncture and related therapies can be considered as an adjunct in patients who experience persistent CRF after they receive first-line treatment. Such application is of high clinical relevance because it is well acknowledged that management of CRF is often suboptimal^35^. Although one SR demonstrated the effectiveness of acupuncture in the management of chronic pain^36^, we identified conflicting results on the effectiveness of acupuncture and related therapies in the management of CRP. Conflicting results were also found in the treatment of hot flashes and hiccups and in the improvement of QoL. No adequately powered, well-designed RCT has been performed to clarify the potential role of acupuncture and related therapies in the management of xerostomia, dyspnea, lymphedema and psychological well-being. Future trials should focus on these under-investigated areas.

Although the application of acupuncture and related therapies in the management of CRF, CINV and leucopenia is supported by results from RCTs, the reporting quality of these studies is often poor, which hindered us from assessing the RoB in these RCTs, and future confirmatory trials should adhere to CONSORT recommendations for reporting. Researchers should also adopt a comparative effectiveness approach and design trials that allow real-world evaluation of acupuncture and related therapies. Specifically, the combined effects of acupuncture and related therapies in addition to guideline-recommended conventional care (e.g., glucocorticoids, 5-HT_3_ antagonists and/or NK_1_R antagonists in CINV) should be compared with conventional care alone so that the additional benefits of acupuncture can be elucidated^37^. Although this approach can make the choice of control intervention more straightforward, the design of a treatment protocol for acupuncture and related therapies is more challenging because it includes a wide variety of techniques^38^. Different researchers may use different techniques, and in other cases the treatment protocol can be individualised according to Chinese medicine diagnostic principles. Such variation is reflected in the included SRs–many adopted a less-restrictive approach and embraced various techniques^6^,^7^,^8^,^9^,^14^,^18^,^20^,^21^,^22^,^23^,^25^, whereas others focused only on a single technique such as moxibustion^31^ and TENS^13^. As a result, the meta-analyses often pooled trials with highly heterogeneous acupuncture and control interventions^14^,^23^, which makes interpretation of their results very difficult. Network meta-analysis^39^ could be a solution that takes into account the heterogeneity amongst treatments in both groups. Unfortunately, the exact intervention protocols used in trials are often not reported^31^, which prevented us from performing such an analysis. Finally, the interpretability of the reported results is also limited because of the wide variations in the choice of outcome measures. For example, pain was measured with a visual analogue scale in some primary studies, but in others it was reported as a binary response rate^22^. Future trials should choose the most clinically relevant endpoint as the primary outcome^40^ and measure it using a validated method^11^ so as to ensure the utility of future clinical evidence^41^.

When deciding on the scope of this overview, we tried to balance the need to reduce heterogeneity at the intervention level on the one hand and the need to ensure appropriate reflection of real clinical practice on the other. Although focusing exclusively on needle acupuncture may facilitate understanding of the effects of a single style of acupuncture, failure to report results from other related therapies may also reduce the comprehensiveness of this overview^42^. From a pragmatic perspective, a combination of needle acupuncture and related therapies such as moxibustion^43^ or TENS^44^ is often used in everyday clinical practice. In addition to treatment heterogeneity, the inclusion criteria in the original study design amongst SRs also requires a balance between reporting only the best available evidence from RCTs and taking a more inclusive approach to ensure comprehensiveness. Although the RCT is the best study design to provide evidence on effectiveness, the results from studies with weaker designs may inform the planning of future RCTs^45^, of which both are valuable from research and clinical practice perspectives. To make this overview informative for clinicians and researchers, we set broad inclusion criteria so that all available evidence on needle acupuncture and related therapies was presented^46^,^47^, but the data are separated according to the type of treatment and study design.

The methodological quality of the SRs included in this overview was satisfactory. Good performance in the following areas was noted: conducting duplicate study selection and data extraction, implementing comprehensive literature search, assessing the scientific quality of included studies and appropriately incorporating scientific quality in the formulation of conclusions. At the same time, improvements should be made in the following areas.

- (i)  The provision of an a priori design for the SRs would reduce the potential bias from reviewers, increase the SR’s transparency and prevent duplicate work on the same topic^10^.
- (ii)  Listing both included and excluded studies would ensure comprehensive reporting of SRs and allow readers to examine the excluded studies if they had a different perspective on eligibility criteria^10^.
- (iii)  Assessing the publication bias of included studies would inform readers on whether the results were generated from an unbiased sample of related studies^10^.
- (iv)  Clarifying conflicts of interest is important because it is well known that financial support might introduce bias to the SR process^48^.

In conclusion, acupuncture and related therapies have demonstrated favourable therapeutic effects in the management of CRF, CINV and leucopenia in cancer patients. Conflicting evidence exists for the treatment of CRP, hot flashes and hiccups and in the improvement of QoL. The currently available evidence is insufficient to support or refute the potential of acupuncture and related therapies in the management of xerostomia, dyspnea and lymphedema and in the improvement of psychological well-being. No serious adverse effects were reported in the included studies, consistent with the results of previous SRs that concluded that acupuncture is a relatively safe treatment option^49^,^50^. Future comparative effectiveness research in this area should pay attention to:

- (i)  improving the reporting and methodological quality of trials;
- (ii)  developing an acupuncture treatment protocol using an expert consensus technique, taking into account regulatory requirements and the constraints of the practice setting;
- (iii)  describing the treatment protocol according to the STRICTA^51^ and TIDieR checklist^52^, so that the procedure can be replicated in other trials or be adopted into clinical practice if it is found to be effective; and
- (iv)  choosing guideline-recommended treatment for the control group and validating outcome measures.

## Methods

### Criteria for considering reviews for inclusion

The Cochrane handbook version 5.1.0.^10^ states that an SR should aim to ‘identify, appraise and synthesise all the empirical evidence that meets pre-specified eligibility criteria to answer a given research question’. Following this definition, we judged a publication as an SR if it sought to answer an explicit clinical question by examining evidence from at least two electronic databases. In this overview, any SRs (including both Cochrane SRs and non-Cochrane SRs) that summarised clinical evidence on the effectiveness of acupuncture and related therapies for palliative care of cancer were considered eligible. If an SR summarised evidence on multiple palliative care techniques, those that reported evidence on acupuncture and related therapies separately were also included.

### Participants

To be eligible, the SRs had to include clinical trials that recruited patients with a diagnosis of any type of cancer who have received acupuncture and related therapies for supportive or palliative care.

### Interventions & control treatments

Any form of acupuncture was considered in this overview. We included SRs that summarised evidence on invasive acupuncture (including needle acupuncture, electro-acupuncture and auricular acupuncture) and non-invasive techniques such as laser acupuncture or acupressure. We also included acupoint injection, moxibustion and TENS. For control treatments, we included SRs that summarised studies that included any type of intervention without acupuncture or the related treatments described above. These interventions included conventional treatment, behavioural therapy, Chinese herbal medicine treatment, sham acupuncture, addition to a waiting list or no treatment.

### Outcomes of interest

We summarised all reported cancer-related or treatment-related outcomes in this overview to provide a comprehensive picture of the available clinical evidence on acupuncture and related therapies for palliative care of cancer.

### Literature search

We searched four international databases (MEDLINE, EMBASE, the Cochrane Database of Systematic Reviews and the Database of Abstracts of Reviews of Effect ) and three Chinese databases (Chinese Biomedical Databases, Wan Fang Digital Journals and Taiwan Periodical Literature Databases) from their inception through July 2014 to identify potential SRs. For MEDLINE and EMBASE, a specialised search filter for SR articles was used^53^,^54^. Comprehensive searches of each database with a full Boolean search strategy were conducted, and the details are reported in Appendix 1.

### Literature selection, data extraction and methodological quality assessment

The citations retrieved from the databases were independently screened and assessed for eligibility by two researchers (XY, RH); disagreements were resolved by discussion and consensus. If a disagreement could not be resolved, a third reviewer (VC) was invited to make a judgment. For duplicate publications, the most updated version was selected.

The following data were extracted from each eligible SR: (i) basic characteristics, including eligibility criteria, designs of included studies, number of included studies, total number of patients and bibliographic information; (ii) details on patients, interventions, controls and outcomes; (iii) synthesised results, including pooled summary effects of each comparison for each outcome if meta-analysis were conducted.

The methodological quality of the included SRs was assessed with the validated Methodological Quality of Systematic Reviews (AMSTAR)^55^ instrument. A detailed operational guide for AMSTAR can be found in Appendix 2. Data extraction and methodological quality assessment were conducted independently by two researchers; disagreements were resolved by discussion and consensus. Any unresolved discrepancies were judged by a third reviewer.

### Data synthesis

The clinical evidence reported in the included SRs was synthesised narratively under each outcome. The effects on dichotomous data were summarised with the pooled RR, which measures the risk of a certain outcome in the treatment group as compared to the risk in the control group across trials. The pooled SMD or WMD was used for continuous outcomes. The SMD was used if a variety of measurement methods were used for the same outcome, and the WMD was used when the outcome was measured with the same method. The protocol of this overview has been registered in PROSPERO (http://www.crd.york.ac.uk/PROSPERO/DisplayPDF.php?ID=CRD42014015576).

## Additional Information

**How to cite this article**: Wu, X. *et al.* Effectiveness of acupuncture and related therapies for palliative care of cancer: overview of systematic reviews. *Sci. Rep.* **5**, 16776; doi: 10.1038/srep16776 (2015).

## Supplementary Material

Supplementary Information - Appendix

## Figures and Tables

**Table 1:** Characteristics of included systematic reviews of RCT on needle acupuncture for cancer palliative care.

Firstauthor andyear ofpublication	Includedstudydesign	Diagnosis	Searchperiod	Nature of acupuncture and relatedinterventions	Nature of controlinterventions	Outcomes	No. ofstudies(No. of patients)included	Meta-analysisconducted?
Lee, 2009b	OnlyRCT	Breastcancer	Aug.2008	Acupuncture with needle insertion: needle acupuncture or electro-acupuncture. Related therapies including laser acupuncture and moxibustion were excluded.	No restriction on type of control. Controls included sham acupuncture, conventional care or relaxation.	Hot flushes.	6 (202)	Yes
Peng, 2010	OnlyRCT	Various	Jun.2008	Needle acupuncture, electro-acupuncture, or auricular acupuncture.	No restriction on type of control. Controls included conventional care, sham acupuncture or no treatment.	Cancer related pain.	7 (634)	No
Pu, 2010	OnlyRCT	Various	Jun.2009	Needle acupuncture or electro-acupuncture.	Conventional care.	Nausea, vomiting and treatment related gastrointestinal adverse reaction.	6 (461)	Yes
Choi, 2012a	OnlyRCT	Various	Apr.2011	Needle acupuncture, auricular acupuncture, electro-acupuncture or “fire needle”.	Sham acupuncture or conventional care.	Cancer related pain, operation related pain.	15 (1157)	Yes
Hurlow, 2012	OnlyRCT	Various	Oct.2010	Single or dual channel TENS	Placebo or placebo plus conventional care.	Cancer related pain.	3 (88)	No
Paley, 2012	OnlyRCT	Various	Jun.2012	Penetrating acupuncture: including needle acupuncture or auricular acupuncture.	Sham acupuncture or conventional care.	Cancer related pain.	3 (204)	No
Posadzki, 2013	OnlyRCT	Various	Nov.2012	Needle acupuncture plus electro-acupuncture; or needle acupuncture plus education.	No restriction on type of control. Controls included sham acupuncture or conventional care.	Cancer-related fatigue	7 (548)	No
Zeng, 2013	OnlyRCT	Various	May2013	Needle acupuncture. Trials using acupuncture without needle insertion was excluded.	Sham acupuncture, conventional care, self- acupressure, no treatment or waiting list.	Cancer-related fatigue, quality of life, functional well-being	7 (689)	Yes
Lian, 2014	OnlyRCT	Various	Jun.2010	Needle acupuncture or electro-acupuncture	Chemotherapy, conventional care or sham acupuncture	Vomiting, abdominal discomfort, diarrhea, peripheral neuropathy; cancer pain, post-operative urinary retention, quality of life, vasomotor syndrome, recovery of gastrointestinal function.	33 (2503)	No

Keys: RCT, randomized controlled trial; TENS, Transcutaneous electrical nerve stimulation.

**Table 2:** Characteristics of included systematic reviews of RCT on acupuncture and related therapies for cancer palliative care.

First authorand year ofpublication	Includedstudy design	Diagnosis	Searchperiod	Nature of acupunctureand relatedinterventions	Nature ofcontrolinterventions	Outcomes	No. of studies(No. of patients)included	Meta- analysisconducted?
Chen, 2013	Only RCT	Lung cancer	Jun. 2013	Needle acupuncture, acupoints injection, moxibustion, and applications of acupoint plaster or magnet.	No restriction on type of control. Controls included conventional care or CHM alone.	Various outcomes, including nausea and vomiting, tumor response, quality of life (measured by Karnofsky performance status & EORCT-QLQ-C30).	31 (1758)	Yes
Lee, 2014	Only RCT	Various	Apr. 2013	Moxibustion (direct, indirect, heat-sensitive, moxa burner, or natural moxibustion).	Conventional care.	Cancer-related fatigue	4 (374)	Yes
Cheon, 2014	Only RCT	Various	Mar. 2013	Acupoints injection with Chinese herbal extract solution; or with conventional medications.	Conventional care	Cancer related pain, chemotherapy-induced nausea and vomiting, lleus, hiccup, fever, quality of life and gastrointestinal symptoms	22 (2459)	Yes
Ezzo, 2014	Only RCT	Various,	Not reported	Needle acupuncture; acupressure; electro-acupuncture or TENS.	No restriction on type of control. Controls included conventional care, sham acupuncture, or conventional care plus sham acupuncture.	Chemotherapy-induced nausea or vomiting, or both.	11 (1247)	Yes

Keys: CHM: Chinese herbal medicine; RCT, randomized controlled trial; TENS, Transcutaneous electrical nerve stimulation.

**Table 3:** Characteristics of included systematic reviews of various study design on acupuncture and related therapies for cancer palliative care.

First authorand year ofpublication	Includedstudy design	Diagnosis	Searchperiod	Nature ofacupuncture andrelated interventions	Nature ofcontrolinterventions	Outcomes	No. of studies(No. of patients)included	Meta analysisconducted?
Lee, 2005	Quasi-RCT	Various	Feb. 2004	Needle acupuncture, ear acupuncture or electro-acupuncture. Other related therapies including laser acupuncture, acupressure, moxibustion and TENS were not reviewed.	Conventional care or sham acupuncture.	Cancer related pain, operation related pain.	7 (368)	No
Lu, 2007	RCT or quasi-RCT	Various	2004	Needle acupuncture, electro-acupuncture with warming needle or acupuncture point injection with saline.	Chemotherapy alone or chemotherapy with vitamins or non-herbal supplements	Leukocytes level.	11 (960)	Yes
Lee, 2009a	RCT, quasi-RCT, or observational studies	Prostate cancer	Dec. 2008	Needle acupuncture, electro-acupuncture or auricular acupuncture.	Conventional care.	Hot flushes.	6 (132)	No
Chao, 2009	RCT, quasi-RCT, or case series study	Breast cancer	Oct. 2008	Needle acupuncture, electro-acupuncture, acupoints injection, self-acupressure or acupoints stimulation by devices.	No restriction on type of control. Controls included placebo, conventional care or no treatment.	Cancer therapy-related adverse events: hot flashes, nausea and vomiting, lymphedema, leukopenia.	26 (1548)	No
Dos Santos, 2010	RCT and case series	Breast cancer	Apr. 2009	Needle acupuncture, electro-acupuncture, acupuncture plus acupressure or auricular acupuncture.	No restriction on type of control. Controls included sham acupuncture, conventional care, waiting list or no treatment.	Cancer therapy-related adverse events: hot flashes, fatigue, pain, dyspnea, psychological well-being, lymphedema and vomiting.	12 (612)	No
O’Sullivan, 2011	RCTs and SR of RCTs	Head and neck cancer	2010	Needle acupuncture or electro-acupuncture. ‘Non-needling’ techniques including laser acupuncture, acupressure or acupuncture-like TENS were not considered.	Sham acupuncture or conventional care.	Irradiation-induced xerostomia	3 (123)	No
Choi, 2012b	RCT and quasi-RCT	Various	Jul. 2011	Needle acupuncture or electro-acupuncture. Other related therapies, including laser acupuncture, acupressure, auricular acupuncture using pressure device, acupoints injection and moxibustion were not considered.	Conventional care.	Cancer related hiccups.	5 (296)	Yes
Finnegan-John, 2013	RCT and quasi-RCT	Various	Jun. 2012	Needle acupuncture or acupressure.	Sham acupuncture.	Cancer-related fatigue	1 (35)	No
Zheng, 2014	RCT and quasi-RCT	Various	2013	Needle acupuncture alone or needle acupuncture plus conventional care.	Conventional care alone.	Cancer related pain	5 (395)	Yes
Frisk, 2014	RCT and case series	Various	Oct. 2012	Needle acupuncture, electro-acupuncture or auricular acupuncture.	No restriction on type of control. Controls included conventional care, sham acupuncture or no treatment.	Hot flushes.	17 (599)	No

Keys: RCT, randomized controlled trial; SR, systematic review; TENS, Transcutaneous electrical nerve stimulation.

**Table 4:** Methodological quality of included systematic reviews on acupuncture and related treatment for cancer palliative care.

First author andpublication year	AMSTAR item										
	1	2	3	4	5	6	7	8	9	10	11
Lee, 2005	N	Y	Y	N	Y	N	Y	N	NA	N	N
Lu, 2007	N	Y	Y	Y	Y	Y	Y	Y	Y	N	N
Lee, 2009a	N	Y	Y	Y	N	Y	Y	Y	NA	N	N
Lee, 2009b	N	Y	Y	Y	N	Y	Y	Y	Y	N	N
Chao, 2009	N	Y	Y	N	N	Y	Y	Y	NA	N	N
Peng, 2010	N	Y	Y	Y	N	N	Y	Y	NA	N	N
Dos Santos, 2010	N	Y	Y	NR	N	Y	Y	Y	NA	N	N
Pu, 2010	N	Y	Y	NR	N	N	Y	Y	Y	N	N
O’Sullivan, 2010	Y	NR	Y	Y	Y	Y	Y	Y	NA	N	N
Choi, 2012a	N	Y	Y	Y	N	Y	Y	Y	Y	N	N
Hurlow, 2012	Y	NR	Y	NR	Y	Y	Y	Y	NA	N	N
Paley, 2012	Y	NR	Y	Y	Y	Y	Y	Y	NA	N	N
Choi, 2012b	N	Y	Y	Y	N	Y	Y	Y	Y	N	N
Posadzki, 2013	N	Y	Y	Y	N	N	Y	Y	NA	N	N
Zeng, 2013	N	Y	Y	N	N	N	Y	Y	Y	N	N
Finnegan-John, 2013	N	Y	Y	N	N	Y	Y	Y	NA	N	N
Chen, 2013	N	Y	Y	Y	N	N	Y	N	N	N	N
Zheng, 2014	N	Y	Y	NR	N	N	Y	Y	Y	N	N
Lee, 2014	Y	Y	Y	Y	N	Y	Y	Y	Y	N	N
Frisk, 2014	N	NR	Y	N	N	N	N	N	NA	N	N
Cheon, 2014	N	N	Y	Y	N	N	Y	Y	Y	N	N
Ezzo, 2014	Y	Y	Y	Y	Y	N	Y	N	N	N	N
Lian, 2014	N	Y	Y	N	N	N	Y	N	NA	N	N
# of Yes (%)	5 (21.7)	18 (78.3)	23 (100.0)	13 (56.5)	6 (26.1)	12 (52.2)	22 (95.6)	18 (78.3)	9 (39.1)	0 (0.0)	0 (0.0)

Keys: N, no; NA, not applicable; NR, not reported; Y, yes (SR fulfilling the criteria); # of Yes, number of yes; AMSTAR item: 1. Was an ‘a priori’ design provided? 2. Was there duplicate study selection and data extraction? 3. Was a comprehensive literature search performed? 4. Was the status of publication (i.e. grey literature) used as an inclusion criterion? 5. Was a list of studies (included and excluded) provided? 6. Were the characteristics of the included studies provided? 7. Was the scientific quality of the included studies assessed and documented? 8. Was the scientific quality of the included studies used appropriately in formulating conclusions? 9. Were the methods used to combine the findings of studies appropriate? 10. Was the likelihood of publication bias assessed? 11. Was the conflict of interest included?

**Table 5:** Clinical evidence on the effectiveness of needle acupuncture on cancer palliative care related symptoms-evidence from SR of RCT.

First author and publication year	Outcome assessment method	Main results	Notes on interpretation
***Cancer related pain***			
Peng, 2010	Total response rate or VAS	One RCT with low RoB reported that auricular acupuncture provides statistically significant relief on CRP when compared to sham acupuncture on Day 30 (p = 0.02) and Day 60 (p < 0.001).	This SR included the same low RoB RCT as Lee 2005 did. All the other six studies suggested positive effect of acupuncture in reducing CRP, although they are judged to have high RoB according to the Jadad scale. Five out of the six studies used total response rate as the primary outcome, which was not a validated outcome.
Choi, 2012a	Validated scales or VAS	Acupuncture showed positive add-on effect on response rate when compared to conventional care alone. No significant differences were found in the comparisons of acupuncture versus conventional care on response rate, or acupuncture versus sham acupuncture on pain score.	Considerable heterogeneity (I^2^ > = 67%) was observed in all three meta-analyses. One study was the same low RoB trial identified by Lee, 2005. All the other trials had poor reporting quality, and were judged as having unclear RoB.
Hurlow, 2012	Validated scales	When comparing TENS with placebo, the results suggested that TENS may improve bone pain during movement. No superior effect in other pain outcomes when comparing TENS with placebo or sham TENS.	All the three trials had small sample sizes (n = 15, 24 and 49). All of them had either unclear RoB for allocation concealment or blinding of outcome assessment.
Paley, 2012	Validated scales	One RCT with low RoB found that auricular acupuncture provided statistically significant relief on CRP when compared to sham acupuncture on Day 30 (p = 0.02) and Day 60 (p < 0.001).	Evidence from the only well designed RCT indicated effective ness of auricular acupuncture in relieving CRP. The other two studies were non-blinded and had incomplete outcome data.
Lian, 2014	VAS or efficacy rate	Results from all six studies suggested that acupuncture is effective in reducing CRP.	RoB of the six studies were assessed with Jadad scale in this SR, of which all scored 2-3 out of a total of 5. However, rationale for supporting these ratings was not given.
***Cancer related fatigue***			
Posadzki, 2013	Validated scales for measuring fatigue	In the two trials with low RoB, one (n = 29) reported significant reduction in fatigue level at 2 weeks in the needle acupuncture group, as compared with the sham acupuncture group. Another study (n = 23) found no significant difference between the acupuncture and sham acupuncture groups.	Acupuncture may be useful for reducing CRF but both trials were underpowered due to small sample size.
Zeng, 2013	General CRF change score	All four sets of comparison favored acupuncture; however, only one comparison (acupuncture plus education versus conventional care) reached statistically significant difference on general CRF level.	All the comparisons had high heterogeneity (I^2^ values ranged from 65% to 94%, under random effect model.). Three trials had low RoB while the other four trials was judged to have high RoB. One had incomplete outcome data the other three lacked of allocation concealment and blinding.
***Hot Flashes***			
Lee, 2009b	diary or logbooks	Results from meta-analysis suggested that acupuncture is effective in reducing hot flashes frequency during treatment when compared to sham acupuncture. However, such difference was not seen after the treatment.	These studies were judged to have low RoB using the Jadad scale, with scoring ranged from 4 to 5 out of 5. Allocation concealment procedures were properly implemented.
***Nausea and vomiting***			
Pu, 2010	Effective rate	Results from meta-analyses indicate that electro-acupuncture and conventional care had similar effect for reducing CINV.	Clinical evidence was from one single clinical trial with poor reporting quality. RoB of this trial was judged to be unclear.
***Quality of Life***			
Zeng, 2013	Change in general QoL scores	Acupuncture showed no favorable effect in improving QoL when compared to sham acupuncture at 10-week follow-up.	These three studies had low RoB. Considerable heterogeneity was found among the three studies (I^2^ = 92%, random effect model was used).
***Quality of Life***			
Lian, 2014	QoL assessment scales and various indexes	Both studies suggested that acupuncture plus conventional care can significantly improve quality of life in cancer patients compared to conventional care alone.	These two trials were judged to have 2 or 3 scores out of 5 in the Jadad scale. However, no rationale was provided on how these scorings were rated.
***Other symptoms***			
Choi, 2012b	Response rates on reducing hiccup	Results from meta-analysis suggested a favorable effect of acupuncture on response rate for patients’ hiccup as compared to conventional care.	All the three studies had poor reporting quality on RoB related information, and were judged to have unclear RoB.

Keys: CINV, chemotherapy induced nausea and vomiting; CRF, cancer related fatigue; CRP, cancer related pain; MFI, multidimensional fatigue inventory; QoL, quality of life; RCT, randomized controlled trial; RoB, risk of bias; SR, systematic review; TENS, Transcutaneous electrical nerve stimulation; VAS, Visual analog scale for pain.

**Table 6:** Clinical evidence on the effectiveness of acupuncture related therapies on cancer palliative care related symptoms-evidence from SR of RCT.

First author and publication year	Outcome assessment method	Main results	Notes on interpretation
***Cancer related pain***			
Cheon, 2014	Response rate	Controversial results were reported among the eight studies. As compared to conventional care, seven studies suggested benefit of acupoint injection in alleviating CRP. While the other one failed to find any positive effect from acupoint injection.	All included trials had high RoB for blinding, and unclear RoB on allocation concealment. All the included trails used response rate as the primary outcome, which was not a validated instrument.
***Cancer related fatigue***			
Lee, 2014	Response rate	Compared to conventional care alone, combination of moxibustion and conventional care showed favorable effect on response rate for CRF.	All the four trials had poor reporting quality. They were judged as having unclear RoB for both allocation concealment and blinding of outcome assessment. Considerable heterogeneity (I^2^ = 74%) was found in this random effect meta-analysis.
***Nausea and vomiting***			
Chen, 2013	Effective rate in reducing of nausea and vomiting (Grade II-IV)	The occurrence of chemotherapy-induced nausea and vomiting at Grade II-IV was remarkably reduced in the acupuncture plus conventional care when compared to conventional care alone.	The SR authors did not provide details on RoB among the included studies.
Cheon, 2014	Response rate	Acupoints injection is suggested to be more effective than conventional care for CINV.	All the included trials had high RoB for blinding, and unclear RoB on allocation concealment.
Ezzo, 2014	Proportion of patients with vomiting/nausea and Mean nausea/nausea severity	Acupuncture is effective in reducing the proportion of patients experiencing acute vomiting, but not in reducing the mean number of delayed vomiting episode, and in reducing severity of acute or delayed nausea.	The authors mentioned that only trials with low RoB were included, but there are no detailed assessment results on RoB for each of the trials.
***Quality of Life***			
Chen, 2013	QLQ-C30 total score	Acupuncture can improve QoL for lung cancer patients as compared to conventional care.	The SR authors did not provide details on RoB assessment for these two studies.
Cheon, 2014	Responder rate (% in Karnofsky score)	Acupoints injection significantly improved QoL compared to conventional care (responder rate: 50% versus 25%, p < 0.01).	Evidence was reported from one single study (n = 108). Details on RoB of this study were not provided by the SR authors.
***Other symptoms***			
Cheon, 2014	Response rates on reducing hiccup	Both trials reported a higher responder rate in the acupoints injection group as compared to conventional care. Result from one study reached statistically significance while the other did not.	The two studies had unclear RoB for allocation concealment and high RoB for blinding.

Keys: CINV, chemotherapy induced nausea and vomiting; CRF, cancer related fatigue; CRP, cancer related pain; QoL, quality of life; RCT, randomized controlled trial; RoB, risk of bias; SR, systematic review.

**Table 7:** Clinical evidence on the effectiveness of acupuncture and related therapies on cancer palliative care related symptoms-evidence from SR on various types of study design.

First author and publication year	Outcome assessment method	Main results	Notes on interpretation
***Cancer related pain***			
Lee, 2005	VAS or patients’ verbal assessment	One RCT with low RoB found auricular acupuncture provides statistically significant relief on CRP when compared to sham acupuncture on Day 30 (p = 0.02) and Day 60 (p < 0.001).	Evidence from the only well designed RCT indicated the effective ness of auricular acupuncture on relieving CRP. The other studies were either non-blinded (n = 2) or designed as case series (n = 4).
Chao, 2009	VAS	Controversial results were reported among the three studies. Significant positive effect from acupuncture was found in studies using VAS and number of analgesia applied (p < 0.05) as measures for CRP, but such result was not seem on Sedation score (p > 0.05).	RoB of the three studies were assessed with Jadad scale in this SR. No details on each RoB domain were provided to facilitate judgment on the overall trustworthy of evidence.
Dos Santos, 2010	VAS, or validated scales	Evidence from both studies suggested that acupuncture is useful in reducing CRP.	One study was the same low RoB trial identified by Lee, 2005. The other study was judged to have high RoB for lack of allocation concealment and blinding.
Zheng, 2014	Symptoms improvement rate	Insufficient evidence to judge the effectiveness of wrist ankle acupuncture in treating cancer pain.	All the included studies were judged to have high RoB for allocation concealment and blinding. However, no rationale supporting the RoB assessment results were provided. Symptom improvement rate was used as the primary outcome, which was not a validated instrument.
***Cancer related fatigue***			
Dos Santos, 2010	MFI	Needle acupuncture group had significantly higher improvement (36%) when compared to either acupressure group (19%) or sham acupressure (0.6%) group.	This trial was judged to have low RoB by the SR authors.
Finnegan-John, 2013	MFI	This SR identified the same trial as Dos Santos, 2010.	See interpretation on results from Dos Santos 2010.
***Hot Flashes***			
Lee, 2009a	Validated scales or patient diary	One trial with low RoB found both needle acupuncture and needle acupuncture + electro-acupuncture were effective for treating hot flush in prostate cancer patients when compared with baseline. No between group difference was found.	The only low RoB RCT compared needle acupuncture with electro-acupuncture; no other comparison was reported by the SR authors. The other five trials was judged to have high RoB with the Jadad scale, but no rationale were given on how the scoring was given.
Chao, 2009	Self-administrated questionnaires	One trial with low RoB reported significant positive effect of acupuncture in reducing hot flushes when compared with sham acupuncture. However, other low RoB trial did not find any difference between the two groups.	The other five trials were judged as having high RoB using the Jadad scale; of which two of them were case series studies.
Dos Santos, 2010	Daily diary/log records No. of hot flashes	Two trials with low RoB found that acupuncture was more effective in reducing hot flushes when compared to sham acupuncture, although the results from one of them did not reach statistical significance.	Current evidence from two trials with low RoB suggests that acupuncture may be useful in reducing hot flashes in breast cancer patients. However, two of the other three trials had high RoB. One did not implement no allocation concealment or blinding; and the other one was only a case series study.
Frisk, 2014	Not reported	Results indicated that acupuncture treatment can reduce hot flashes in women with breast cancer and men with prostate cancer over a 3 months period. Nevertheless, it is not reported that how the outcome was measured.	The SR authors reported all the seven trial scored ≥3 score out of 5 in the Jadad scale, however, no rationale on the scorings were given.
***Nausea and vomiting***			
Chao, 2009	Validated scale	One trial with low RoB reported positive effect of electro-acupuncture in treating CINV when compared with sham acupuncture or conventional care. The effect persisted for five days, and it was not sustained from ninth days onward.	The remaining 10 trials were judged to have high RoB by the Jadad scale, of which two of them were case series studies. However, no rationale on the scorings was given.
***Nausea and vomiting***			
Dos Santos, 2010	Total no. of vomiting episodes and no. of vomiting free days	Patients in electro-acupuncture group had significantly greater proportion of nausea-and-vomiting-free days than patients in the other two groups during a 5-day treatment period; this benefit did not persist onward to the ninth days follow up. This SR identified the same low RoB trial as in Chao, 2009 above.	This evidence was from one clinical trial with low RoB for allocation concealment and blinding of outcome assessment. However, RoB for blinding of participants and personnel were high.
***Other symptoms***			
Lu, 2007	Leucopenia (change in WBC count	Results from meta-analysis suggested that acupuncture plus conventional care was an effective option for chemotherapy-induced leukopenia as compared to conventional care alone.	All the included studies were judged to be of high RoB under the Jadad scale. However, no rationale was provided on how these scorings were rated.
Chao, 2009	RILP	The authors reported that WBC level were increased but no further details were provided.	The result is reported from a small case series study.
Chao, 2009 & Dos Santos, 2010	BCRL	In both SRs, acupuncture was found to be effective in treating BCRL. It is mentioned that the participants’ body circulation was enhanced, and sense of heaviness was reduced. Patients have also reported significant improvement on the range of movements, including shoulder flexion and abduction of the affected limbs.	The result is reported from a small case series study
Choi, 2012b	Response rates on reducing hiccup	Results from meta-analysis suggested a favorable effect of acupuncture on response rate for patients’ hiccup as compared to conventional care.	All the three studies had poor reporting quality on RoB related information, and were judged to have unclear RoB.
O’Sullivan, 2011	Improvement on Xerostomia as measured by SFR	All three trials reported significant improvement on SFR when compared to baseline (p < 0.05), but no significant differences were seen between acupuncture and sham acupuncture group.	All the three studies used objective outcome measurements to reduce detection bias. One trial has low RoB for allocation concealment while the other two have unclear RoB.
***Other symptoms***			
Dos Santos, 2010	Rating scale on dyspnea.	In both groups, significantly improvement on dyspnea scores were observed immediately after treatment (p = 0.003). Dyspnea scores were slightly higher in acupuncture group than sham group, however, no significant differences were seen.	The trial was judged to have low RoB, but there is a lacking of blinding of personnel.

Keys: BCRL, breast cancer-related lymphedema; CINV, chemotherapy induced nausea and vomiting; CRP, cancer related pain; MFI, multidimensional fatigue inventory; RCT, randomized controlled trial; RILP, radiotherapy induced leukopenia; RoB, risk of bias; SFR, salivary flow rates; SR, systematic review; VAS, Visual analog scale for pain; WBC, white blood cell.
