# Supplementary material for: Effectiveness of acupuncture and related therapies for palliative care of cancer: overview of systematic reviews
Source: Sci Rep. 2015 Nov 26;5:16776. doi: 10.1038/srep16776 (PMC4660374; doi:10.1038/srep16776)
Supplement: Supplementary Information - Appendix [file srep16776-s1.doc]

**Effectiveness of acupuncture and related therapies for palliative care of cancer: overview of systematic reviews**

Xinyin Wu^1,2^, Vincent CH Chung*^1,2^, Edwin P Hui^1,3^, Eric TC Ziea^4^, Bacon FL Ng^4^, Robin ST Ho^2^, Kelvin KF Tsoi^2,5^, Samuel YS Wong^1,2^, Justin CY Wu^1,6^

1. Hong Kong Institute of Integrative Medicine, The Chinese University of Hong Kong, Hong Kong
2. Jockey Club School of Public Health and Primary Care, The Chinese University of Hong Kong, Hong Kong
3. Comprehensive Cancer Trials Unit, The Chinese University of Hong Kong, Hong Kong
4. Chinese Medicine Department, Hong Kong Hospital Authority, Hong Kong
5. Big Data Decision Analytics Research Centre, The Chinese University of Hong Kong, Hong Kong
6. Department of Medicine & Therapeutics, The Chinese University of Hong Kong, Hong Kong

*Corresponding author: Prof. Vincent CH Chung, 4/F, School of Public Health Building, Prince of Wales Hospital, Shatin, New Territories, Hong Kong; phone: (+852) 2252 8453; email: vchung@cuhk.edu.hk

**Appendix 1. Search strategies and results for systematic review on acupuncture for cancer palliative care**

i) [Cochrane Database of Systematic Reviews](http://www.cochrane.org/editorial-and-publishing-policy-resource/cochrane-database-systematic-reviews-cdsr) (CDSR) from inception to 3/9/2014

| 1 | palliative car*.mp. | 166 |
| --- | --- | --- |
| 2 | terminal car*.mp. | 34 |
| 3 | terminal ill*.mp. | 32 |
| 4 | palliat*.mp. | 450 |
| 5 | (terminal* and (car* or ill*)).mp. | 323 |
| 6 | ((advanced or end stage or terminal*) adj4 (diseas* or ill* or cancer* or malignan*)).mp. | 794 |
| 7 | (last year of life or LYOL or life end or end of life).mp. | 95 |
| 8 | 1 or 2 or 3 or 4 or 5 or 6 or 7 | 1252 |
| 9 | acupunctur*.mp. | 414 |
| 10 | electroacupunctur*.mp. | 82 |
| 11 | electro-acupunctur*.mp. | 60 |
| 12 | acupoint*.mp. | 66 |
| 13 | transcutaneous electric nerve stimulat*.mp. | 37 |
| 14 | auriculoacupunctur*.mp. | 2 |
| 15 | percutaneous electrical nerve stimulat*.mp. | 3 |
| 16 | TENS.mp. | 146 |
| 17 | 9 or 10 or 11 or 12 or 13 or 14 or 15 or 16 | 481 |
| **18** | **8 and 17** | **74** |

ii) [Database of Abstracts of Reviews of Effects](https://www.google.com.hk/url?sa=t&rct=j&q=&esrc=s&source=web&cd=1&ved=0CBsQFjAA&url=http%3A%2F%2Fwww.cochrane.org%2Feditorial-and-publishing-policy-resource%2Fdatabase-abstracts-reviews-effects-dare&ei=m64SVPrVJMLc8AWq6YKoDQ&usg=AFQjCNGBBl9NZsprxhJK7D2IyfGPbWeECg&sig2=8WwnLa2zb4_sRMpBoD5AkA&bvm=bv.75097201,d.dGc&cad=rjt) (DARE) from inception to 3/9/2014

| 1 | palliative car*.mp. | 196 |
| --- | --- | --- |
| 2 | terminal car*.mp. | 41 |
| 3 | terminal ill*.mp. | 12 |
| 4 | palliat*.mp. | 269 |
| 5 | (terminal* and (car* or ill*)).mp. | 99 |
| 6 | ((advanced or end stage or terminal*) adj4 (diseas* or ill* or cancer* or malignan*)).mp. | 555 |
| 7 | (last year of life or LYOL or life end or end of life).mp. | 30 |
| 8 | 1 or 2 or 3 or 4 or 5 or 6 or 7 | 803 |
| 9 | acupunctur*.mp. | 422 |
| 10 | electroacupunctur*.mp. | 55 |
| 11 | electro-acupunctur*.mp. | 26 |
| 12 | acupoint*.mp. | 42 |
| 13 | transcutaneous electric nerve stimulat*.mp. | 48 |
| 14 | auriculoacupunctur*.mp. | 0 |
| 15 | percutaneous electrical nerve stimulat*.mp. | 1 |
| 16 | TENS.mp. | 56 |
| 17 | 9 or 10 or 11 or 12 or 13 or 14 or 15 or 16 | 481 |
| **18** | **8 and 17** | **12** |

iii) MEDLINE from inception to 3/9/2014

| 1 | MEDLINE.tw. | 54034 |
| --- | --- | --- |
| 2 | systematic review.tw. | 42879 |
| 3 | meta analysis.pt. | 51267 |
| 4 | 1 or 2 or 3 | 110972 |
| 5 | exp palliative care/ | 41061 |
| 6 | exp terminal care/ | 42274 |
| 7 | exp terminally ill/ | 5865 |
| 8 | palliat*.mp. | 63977 |
| 9 | palliative car*.mp. | 44119 |
| 10 | terminal car*.mp. | 23513 |
| 11 | terminally ill*.mp. | 9003 |
| 12 | (terminal* and (car* or ill*)).mp. | 37400 |
| 13 | ((advanced or end stage or terminal*) adj4 (diseas* or ill* or cancer* or malignan*)).mp. | 118498 |
| 14 | (last year of life or LYOL or life end or end of life).mp. | 11417 |
| 15 | 5 or 6 or 7 or 8 or 9 or 10 or 11 or 12 or 13 or 14 | 211993 |
| 16 | exp acupuncture therapy/ | 16922 |
| 17 | exp acupuncture, ear/ | 266 |
| 18 | exp acupuncture points/ | 4015 |
| 19 | exp acupuncture analgesia/ | 1044 |
| 20 | exp acupuncture/ | 1246 |
| 21 | acupunctur*.mp. | 18257 |
| 22 | exp electroacupuncture/ | 2552 |
| 23 | electroacupunctur*.mp. | 3161 |
| 24 | acupoint*.mp. | 2538 |
| 25 | exp electric stimulation therapy/ | 59813 |
| 26 | exp transcutaneous electric nerve stimulation/ | 5947 |
| 27 | transcutaneous electric nerve stimulat*.mp. | 3503 |
| 28 | electric stimulation therap*.mp. | 17216 |
| 29 | percutaneous electrical nerve stimulat*.mp. | 35 |
| 30 | auriculoacupunctur*.mp. | 9 |
| 31 | TENS.mp. | 7276 |
| 32 | 16 or 17 or 18 or 19 or 20 or 21 or 22 or 23 or 24 or 25 or 26 or 27 or 28 or 29 or 30 or 31 | 82090 |
| **33** | **4 and 15 and 32** | **46** |

iv) EMABSE from inception to 3/9/2014

| 1 | meta-analysis.tw. | 70115 |
| --- | --- | --- |
| 2 | systematic review.tw. | 60452 |
| 3 | 1 or 2 | 111604 |
| 4 | exp palliative therapy/ | 66664 |
| 5 | exp terminal care/ | 47273 |
| 6 | exp terminal disease/ | 4812 |
| 7 | exp terminally ill patient/ | 6249 |
| 8 | exp cancer patient/ | 126211 |
| 9 | palliative therap*.mp. | 67642 |
| 10 | terminal car*.mp. | 26601 |
| 11 | terminally ill patient*.mp. | 7400 |
| 12 | cancer patient*.mp. | 221822 |
| 13 | palliat*.mp. | 96997 |
| 14 | (terminal* and (car* or ill*)).mp. | 215666 |
| 15 | ((advanced or end stage or terminal*) adj4 (diseas* or ill* or cancer* or malignan*)).mp. | 202131 |
| 16 | (last year of life or LYOL or life end or end of life).mp. | 17553 |
| 17 | 4 or 5 or 6 or 7 or 8 or 9 or 10 or 11 or 12 or 13 or 14 or 15 or 16 | 679148 |
| 18 | exp acupuncture analgesia/ | 1300 |
| 19 | exp acupuncture/ | 32981 |
| 20 | exp acupuncture needle/ | 248 |
| 21 | acupunctur*.mp. | 32707 |
| 22 | exp electroacupuncture/ | 4121 |
| 23 | electroacupunctur*.mp. | 4863 |
| 24 | exp electrostimulation/ | 69347 |
| 25 | exp electrostimulation therapy/ | 177236 |
| 26 | electro-acupunctur*.mp. | 872 |
| 27 | acupoint*.mp. | 3720 |
| 28 | exp transcutaneous nerve stimulation/ | 5716 |
| 29 | transcutaneous nerve stimulat*.mp. | 5874 |
| 30 | exp percutaneous electrical nerve stimulation/ | 5716 |
| 31 | percutaneous electrical nerve stimulat*.mp. | 65 |
| 32 | TENS.mp. | 9809 |
| 33 | auriculoacupunctur*.mp. | 13 |
| 34 | 18 or 19 or 20 or 21 or 22 or 23 or 24 or 25 or 26 or 27 or 28 or 29 or 30 or 31 or 32 or 33 | 272909 |
| **35** | **3 and 17 and 34** | **83** |

v) Chinese Biomedical Database (CBM) [Chinese] from inception to 23/7/2014

("系统综述"[全字段] OR "荟萃分析"[全字段] OR "META"[全字段]) AND ("针灸"[全字段] OR "针刺"[全字段] OR "电针"[全字段] OR "耳针"[全字段] OR “头针” [全字段] OR “水针” [全字段]) AND ("肿瘤"[全字段] OR "癌"[全字段]) (yielded 10 citations)

vi) Wan Fang Digital Journals [Chinese] from inception to 23/7/2014

("系统综述" OR "荟萃分析" OR "META") AND ("针灸" OR "针刺" OR "电针" OR "耳针" OR “头针” OR “水针”) AND ("肿瘤" OR "癌") (yielded 7 citations)

vii) Taiwan Periodical Literature Databases [Chinese] from inception to 23/7/2014

(TX=系統綜述 OR薈萃分析 OR META) [AND] (TX=針灸 OR 針刺 OR 電針 OR 耳針 OR 頭針 OR 水針) [AND] (TX=腫瘤 OR 癌 OR 癌症) (yielded 4 citations)

**Appendix 2. Detailed operational guide for applying the AMSTAR tool***

| **Amstar 1. Was an 'a priori' design provided?**  The research question and inclusion criteria should be established before the conduct of the review.  **Operational Definition (OD):** A “Yes” will be given if the review has published a protocol for the systematic review. | - Yes - No |
| --- | --- |
| **Amstar 2. Was there duplicate study selection and data extraction?**  There should be at least two independent data extractors and a consensus procedure for disagreements should be in place.  **OD:** A “Yes” will be given if:  **(i)** Two reviewers performed study selection, AND  **(ii)** Two reviewers performed data extraction AND  **(iii)** Consensus process was implemented for resolving disagreement. | - Yes (i + ii + iii) - No (These 3 criteria were not fulfilled) - Could not answer (i.e. not reported) |
| **Amstar 3. Was a comprehensive literature search performed?**  At least two electronic sources should be searched. The report must include years and databases used (e.g., CENTRAL, EMBASE, and MEDLINE). Key words and/or MESH terms must be stated and where feasible the search strategy should be provided. All searches should be supplemented by consulting current contents, reviews, textbooks, specialized registers, or experts in the particular field of study, and by reviewing the references in the studies found.  **OD:** A “Yes” will be given if at least two electronic sources plus one supplementary strategy were used (e.g. Cochrane register/CENTRAL counts as two sources; a grey literature search counts as supplementary). (SIGLE database, dissertations, conference proceedings, and trial registries are all considered grey ) | - Yes - No - Could not answer (i.e. not reported) |
| **Amstar 4. Was the status of publication (i.e. grey literature) used as an inclusion criterion?**  The authors should state that they searched for reports regardless of their publication type. The authors should state whether or not they excluded any reports from the systematic review, based on their publication status, language etc.  If review indicates that there was a search for “grey literature” or “unpublished literature,” indicate “yes.” SIGLE database, dissertations, conference proceedings, and trial registries are all considered grey for this purpose. If searching a source that contains both grey and non-grey, must specify that they were searching for grey/unpublished literature.  **OD:** If eligibility criterion is restricted to “non-grey” literature, a “No” will be given, which indicates a methodological shortcoming in this domain. | - Yes - No - Could not answer (i.e. not reported) |
| **Amstar 5. Was a list of studies (included and excluded) provided?**  A list of included and excluded studies should be provided.  **OD:** A “Yes” will be given if the included and excluded studies are referenced. | - Yes - No |
| **Amstar 6. Were the characteristics of the included studies provided?**  In an aggregated form such as a table, data from the original studies should be provided on the participants, interventions and outcomes***.*** The ranges of characteristics in all the studies analyzed e.g., age, race, sex, relevant socioeconomic data, disease status, duration, severity, or other diseases should be reported.  **OD:** A “Yes” will be given if the information described above is presented appropriately. | - Yes - No |
| **Amstar 7. Was the scientific quality of the included studies assessed and documented?**  'A priori' methods of assessment should be provided (e.g., the use of Cochrane Risk of Bias tool as a mean to assessment); for other types of studies alternative tools will also be acceptable.  **OD:** To score a “Yes”, the authors should report risk of bias level in each of the methodological domain included in the risk of bias assessment tool that the authors have chosen to use. | - Yes - No |
| **Amstar 8. Was the scientific quality of the included studies used appropriately in formulating conclusions?**  The results of the methodological rigor and scientific quality should be considered in the analysis and the conclusions of the review, and *considerations on how risk of bias among included study may impact conclusion should be explicitly stated*.  **OD:** To score a “Yes”, the reviewers must consider risk of bias explicitly when writing the conclusion section of the MA. For example, a reviewer may state: the results should be interpreted with caution due to high risk of bias among included studies. A “No” will be given if answer to Q7 is “No”. | - Yes - No |
| **Amstar 9. Were the methods used to combine the findings of studies appropriate?^#^**  For meta-analysis, statistical tests should be done to ensure that the studies were combinable by assessing their homogeneity. This can be done by using the Cochran Q test or reporting the I^2^ value.  **OD:** “Yes” will be given when one of the two situation applies:   1. Homogeneity is found, and authors used fixed effect model or random effect model, or 2. Heterogeneity is found, and authors performed appropriate subgroup analysis or meta-regression.   “No” will be given when one of the two situation applies:   1. Heterogeneity is found, and authors used fixed or random effect model and reported the results directly without highlighting the role of heterogeneity; 2. Heterogeneity is not assessed or reported. | - Yes - No - Not applicable (Meta-analysis was not conducted) |
| **Amstar 10. Was the likelihood of publication bias assessed?**  An assessment of publication bias should include a combination of graphical aids (e.g., funnel plot) and/or statistical tests (e.g., Egger regression test).  **OD:** A “No” will be given if no relevant test values or funnel plot was reported. However, a “Yes” will still be given if authors mentioned that publication bias could not be assessed because there were fewer than 10 included studies . | - Yes - No |
| **Amstar 11. Was the conflict of interest included?**  Potential sources of support should be clearly acknowledged in both the systematic review and the included studies.  **OD:** To score a “Yes”, the authors must indicate source of funding or support for the systematic review AND for each of the included studies. A “Yes” will still be scored if the authors acknowledged that funding sources for included randomized controlled trials were unknown. | - Yes - No |

*adapted from the official AMSTAR website (<http://www.amstar.ca/>)

Appendix3. AMSTAR results of the 23 included systematic reviews on acupuncture and related treatment for cancer palliative care


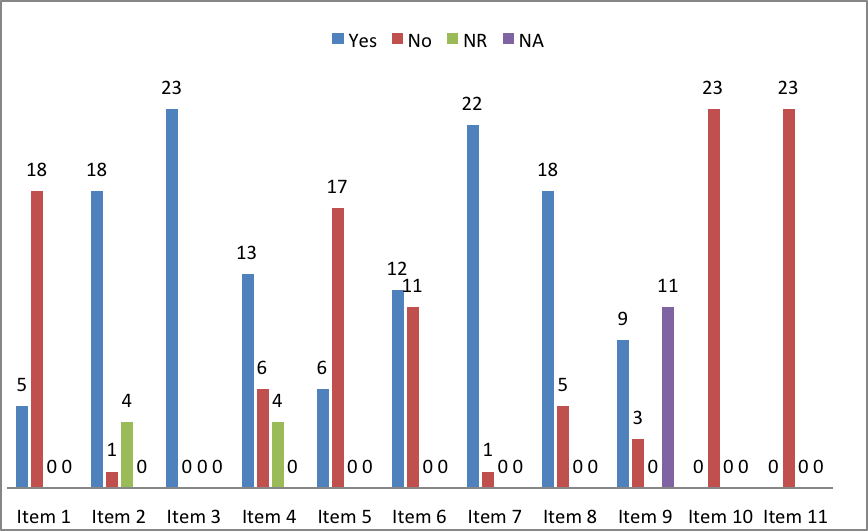


Keys: The number above each bar donated the number of systematic reviews that judged as yes, no, NR or NA for each item. NR, not reported; NA, not applicable; AMSTAR item: 1. Was an 'a priori' design provided? 2. Was there duplicate study selection and data extraction? 3. Was a comprehensive literature search performed? 4. Was the status of publication (i.e. grey literature) used as an inclusion criterion? 5. Was a list of studies (included and excluded) provided? 6. Were the characteristics of the included studies provided? 7. Was the scientific quality of the included studies assessed and documented? 8. Was the scientific quality of the included studies used appropriately in formulating conclusions? 9. Were the methods used to combine the findings of studies appropriate? 10. Was the likelihood of publication bias assessed? 11. Was the conflict of interest included?

|  | | | **Appendix 4. Quantitative Results on the Clinical evidence on the effectiveness of acupuncture and related therapies on cancer palliative care related symptoms** | | | | | | | |
| --- | --- | --- | --- | --- | --- | --- | --- | --- | --- | --- |
| First author and publication year | | Outcome assessment method | | No. of studies (No. of patients) | *Comparison and pooled results (95%CI) | | | Main results | | Notes on interpretation |
| ***Cancer related pain*** | | | | | | | | | | |
| Lee, 2005 | | VAS or patients' verbal assessment | | 7 (368) | No meta-analysis conducted | | | One RCT with low RoB found auricular acupuncture provides statistically significant relief on CRP when compared to sham acupuncture on Day 30 (p=0.02) and Day 60 (p<0.001). | | Evidence from the only well designed RCT indicated the effective ness of auricular acupuncture on relieving CRP. The other studies were either non-blinded (n=2) or designed as case series (n=4). |
| Chao, 2009 | | VAS | | 3 (278) | No meta-analysis conducted | | | Controversial results were reported among the three studies. Significant positive effect from acupuncture was found in studies using VAS and number of analgesia applied (p<0.05) as measures for CRP, but such result was not seem on Sedation score (p>0.05) . | | RoB of the three studies were assessed with Jadad scale in this SR. No details on each RoB domain were provided to facilitate judgment on the overall trustworthy of evidence. |
| Peng, 2010 | | Total response rate or VAS | | 7 (634) | No meta-analysis conducted |  | | One RCT with low RoB reported that auricular acupuncture provides statistically significant relief on CRP when compared to sham acupuncture on Day 30 (p=0.02) and Day 60 (p<0.001). | | This SR included the same low RoB RCT as Lee 2005 did. All the other six studies suggested positive effect of acupuncture in reducing CRP, although they are judged to have high RoB according to the Jadad scale. Five out of the six studies used total response rate as the primary outcome, which was not a validated outcome. |
| Dos Santos, 2010 | | VAS, or validated scales | | 2 (111) | No meta-analysis conducted | | | Evidence from both studies suggested that acupuncture is useful in reducing CRP. | | One study was the same low RoB trial identified by Lee, 2005. The other study was judged to have high RoB for lack of allocation concealment and blinding. |
| Choi, 2012a | | Binary response rate on CRP reduction | | 8 (886) | Acupuncture versus conventional care: RR= 1.12(0.98, 1.28) | | | Acupuncture showed positive add-on effect on response rate when compared to conventional care alone. No significant differences were found in the comparisons of acupuncture versus conventional care on response rate, or acupuncture versus sham acupuncture on pain score. | | Considerable heterogeneity (I^2^ >=67%) was observed in all three meta-analyses. One study was the same low RoB trial identified by Lee, 2005. All the other trials had poor reporting quality, and were judged as having unclear RoB. |
|  |  | Binary response rate on CRP reduction | | 7 (437) | Acupuncture + conventional care versus conventional care: RR=1.36(1.13, 1.64) | | |  |  |  |
|  |  | Validated scales or VAS | | 2 (79) | Acupuncture versus sham Acupuncture:  SMD= -0.41(-1.32, 0.95) | | |  |  |  |
| Hurlow, 2012 | | Validated scales | | 3 (88) | No meta-analysis conducted | | | When comparing TENS with placebo, the results suggested that TENS may improve bone pain during movement. No superior effect in other pain outcomes when comparing TENS with placebo or sham TENS. | | All the three trials had small sample sizes (n=15, 24 and 49). All of them had either unclear RoB for allocation concealment or blinding of outcome assessment. |
| Paley, 2012 | | Validated scales | | 3 (204) | No meta-analysis conducted | | | One RCT with low RoB found that auricular acupuncture provided statistically significant relief on CRP when compared to sham acupuncture on Day 30 (p=0.02) and Day 60 (p<0.001). | | Evidence from the only well designed RCT indicated effective ness of auricular acupuncture in relieving CRP. The other two studies were non-blinded and had incomplete outcome data. |
| Zheng, 2014 | | Symptoms improvement rate | | 5 (395) | Wrist ankle acupuncture + conventional care versus conventional care:  RR= 1.12 (0.92, 1.36) | | | Insufficient evidence to judge the effectiveness of wrist ankle acupuncture in treating cancer pain. | | All the included studies were judged to have high RoB for allocation concealment and blinding. However, no rationale supporting the RoB assessment results were provided. Symptom improvement rate was used as the primary outcome, which was not a validated instrument. |
| Cheon, 2014 | | Response rate | | 8 (680) | No meta-analysis conducted | | | Controversial results were reported among the eight studies. As compared to conventional care, seven studies suggested benefit of acupoint injection in alleviating CRP. While the other one failed to find any positive effect from acupoint injection. | | All included trials had high RoB for blinding, and unclear RoB on allocation concealment. All the included trails used response rate as the primary outcome, which was not a validated instrument. |
| Lian, 2014 | | VAS or efficacy rate | | 6 (490) | No meta-analysis conducted | | | Results from all six studies suggested that acupuncture is effective in reducing CRP. | | RoB of the six studies were assessed with Jadad scale in this SR, of which all scored 2-3 out of a total of 5. However, rationale for supporting these ratings was not given. |
| ***Cancer related fatigue*** | | | | | | | | | | |
| Dos Santos, 2010 | Multidimensional fatigue inventory (MFI) | | | 1 (47) | No meta-analysis conducted | | Needle acupuncture group had significantly higher improvement (36%) when compared to either acupressure group (19%) or sham acupressure (0.6%) group. | | This trial was judged to have low RoB by the SR authors. | |
| Posadzki, 2013 | Validated scales for measuring fatigue | | | 7 (548) | No meta-analysis conducted |  | In the two trials with low RoB, one (n=29) reported significant reduction in fatigue level at 2 weeks in the needle acupuncture group, as compared with the sham acupuncture group. Another study (n=23) found no significant difference between the acupuncture and sham acupuncture groups. | | Acupuncture may be useful for reducing CRF but both trials were underpowered due to small sample size. | |
| Zeng, 2013 | General CRF change score | | | 3 (121) | Acupuncture versus sham acupuncture :  SMD = -0.82(-1.90, 0.26) |  | All four sets of comparison favored acupuncture; however, only one comparison (acupuncture plus education versus conventional care) reached statistically significant difference on general CRF level. | | All the comparisons had high heterogeneity (I^2^ values ranged from 65% to 94%, under random effect model.). Three trials had low RoB while the other four trials was judged to have high RoB. One had incomplete outcome data the other three lacked of allocation concealment and blinding. | |
|  | General CRF change scores | | | 2 (314) | Acupuncture plus education versus conventional care :  SMD= -2.12(-3.21, -1.03) |  |  |  |  |  |
|  | General CRF change scores | | | 2 (150) | Acupuncture versus no treatment/ waiting-list: SMD= -1.46(-3.56, 0.63) |  |  |  |  |  |
|  | General CRF change scores | | | 2 (163) | Acupuncture versus acupressure/self-acupuncture:  SMD= -1.12(-3.03, 0.78) |  |  |  |  |  |
| Finnegan-John, 2013 | MFI | | | 1 (47) | No meta-analysis conducted |  | This SR identified the same trial as Dos Santos, 2010. | | See interpretation on results from Dos Santos 2010. | |
| Lee, 2014 | Response rate | | | 4 (340) | Moxibustion + conventional care versus conventional care alone: SMD=1.73 (1.29, 2.32) |  | Compared to conventional care alone, combination of moxibustion and conventional care showed favorable effect on response rate for CRF. | | All the four trials had poor reporting quality. They were judged as having unclear RoB for both allocation concealment and blinding of outcome assessment. Considerable heterogeneity (I^2^=74%) was found in this random effect meta-analysis. | |
| ***Hot Flashes*** | | | | | | | | | | |
| Lee, 2009a | Validated scales or patient diary | | | 6 (132) | No meta-analysis conducted | | One trial with low RoB found both needle acupuncture and needle acupuncture + electro-acupuncture were effective for treating hot flush in prostate cancer patients when compared with baseline. No between group difference was found. | | The only low RoB RCT compared needle acupuncture with electro-acupuncture; no other comparison was reported by the SR authors. The other five trials was judged to have high RoB with the Jadad scale, but no rationale were given on how the scoring was given. | |
| Lee, 2009b | Hot flashes after treatment: diary or logbooks | | | 3 (189) | Acupuncture versus sham acupuncture:  SMD=3.09 (-0.04, 6.23) | | Results from meta-analysis suggested that acupuncture is effective in reducing hot flashes frequency during treatment when compared to sham acupuncture. However, such difference was not seen after the treatment. | | These studies were judged to have low RoB using the Jadad scale, with scoring ranged from 4 to 5 out of 5. Allocation concealment procedures were properly implemented. | |
|  | Hot flashes during treatment: diary or logbooks | | | 3 (189) | Acupuncture versus sham acupuncture:  SMD=1.91 (0.10, 3.71) | |  |  |  |  |
| Chao, 2009 | Self-administrated questionnaires | | | 7 (281) | No meta-analysis conducted | | One trial with low RoB reported significant positive effect of acupuncture in reducing hot flushes when compared with sham acupuncture. However, other low RoB trial did not find any difference between the two groups. | | The other five trials were judged as having high RoB using the Jadad scale; of which two of them were case series studies. | |
| Dos Santos, 2010 | Daily diary/log records No. of hot flashes | | | 5 (236) | No meta-analysis conducted | | Two trials with low RoB found that acupuncture was more effective in reducing hot flushes when compared to sham acupuncture, although the results from one of them did not reach statistical significance. | | Current evidence from two trials with low RoB suggests that acupuncture may be useful in reducing hot flashes in breast cancer patients. However, two of the other three trials had high RoB. One did not implement no allocation concealment or blinding; and the other one was only a case series study. | |
| Frisk, 2014 | Not reported | | | 7 (472) | No meta-analysis conducted | | Results indicated that acupuncture treatment can reduce hot flashes in women with breast cancer and men with prostate cancer over a 3 months period. Nevertheless, it is not reported that how the outcome was measured. | | The SR authors reported all the seven trial scored ≥3 score out of 5 in the Jadad scale, however, no rationale on the scorings were given. | |
| ***Nausea and vomiting*** | | | | | | | | | | |
| Chao, 2009 | Validated scale | | | 11 (878) | No meta-analysis conducted |  | One trial with low RoB reported positive effect of electro-acupuncture in treating CINV when compared with sham acupuncture or conventional care. The effect persisted for five days, and it was not sustained from ninth days onward. | | The remaining 10 trials were judged to have high RoB by the Jadad scale, of which two of them were case series studies. However, no rationale on the scorings was given. . | |
| Dos Santos, 2010 | Total no. of vomiting episodes and no. of vomiting free days | | | 1 (104) | No meta-analysis conducted | | Patients in electro-acupuncture group had significantly greater proportion of nausea-and-vomiting-free days than patients in the other two groups during a 5-day treatment period; this benefit did not persist onward to the ninth days follow up. This SR identified the same low RoB trial as in Chao, 2009 above. | | This evidence was from one clinical trial with low RoB for allocation concealment and blinding of outcome assessment. However, RoB for blinding of participants and personnel were high. | |
| Pu, 2010 | Effective rate in reducing nausea | | | 1 (78) | Electro-acupuncture versus Metoclopramide: RR=3.13(0.99, 9.90) |  | Results from meta-analyses indicate that electro-acupuncture and conventional care had similar effect for reducing CINV. | | Clinical evidence was from one single clinical trial with poor reporting quality. RoB of this trial was judged to be unclear. | |
|  | Effective rate in reducing nausea | | | 1 (78) | Electroacupuncture versus Ondansetron:  RR=0.78(0.43, 1.43) | |  |  |  |  |
|  | Effective rate in reducing vomiting | | | 1 (78) | Electroacupuncture versus Metoclopramide:  RR=1.88(0.97, 3.62) | |  |  |  |  |
|  | Effective rate in reducing vomiting | | | 1 (78) | Electroacupuncture versus Ondansetron:  RR=1.15(0.07, 1.90) | |  |  |  |  |
| Chen, 2013 | Effective rate in reducing of nausea and vomiting (Grade II-IV) | | | 8 (501) | Needle acupuncture/ acupoint injection/plaster acupuncture/moxibustion + conventional care versus conventional care alone:  RR=0.46 (0.37, 0.57) | | The occurrence of chemotherapy-induced nausea and vomiting at Grade II-IV was remarkably reduced in the acupuncture plus conventional care when compared to conventional care alone. | | The SR authors did not provide details on RoB among the included studies. | |
| Cheon, 2014 | Response rate in CINV severity reduction | | | 3 (270) | Acupoints injection versus intravenous injection  RR=1.28 (1.14, 1.44) | | Acupoints injection is suggested to be more effective than conventional care for CINV. | | All the included trials had high RoB for blinding, and unclear RoB on allocation concealment. | |
|  | Response rate in CINV frequency reduction | | | 2 (509) | Acupoints injection versus intravenous injection:  RR=2.47 (2.12, 2.89) | |  |  |  |  |
| Ezzo, 2014 | Proportion of patients with vomiting in first 24 hours | | | 9 (1214) | Needle acupuncture/electro-acupuncture/acupressure versus control (including conventional care or sham acupuncture):  RR=0.82 (0.69, 0.99) | | Acupuncture is effective in reducing the proportion of patients experiencing acute vomiting, but not in reducing the mean number of delayed vomiting episode, and in reducing severity of acute or delayed nausea. | | The authors mentioned that only trials with low RoB were included, but there are no detailed assessment results on RoB for each of the trials. | |
|  | Mean nausea severity in first 24 h | | | 7 (896) | Needle acupuncture/electro-acupuncture/acupressure versus control (all types): SMD=-0.11 (-0.25, 0.02) | |  |  |  |  |
|  | Mean no. of vomiting episodes day 2 - day 5-7 | | | 3 (508) | Needle acupuncture/electro-acupuncture/acupressure versus control (all types): SMD=0.02 ( -0.13, 0.17) | |  |  |  |  |
|  | Mean nausea severity day 2 - day 5-7 | | | 3 (821) | Need acupuncture/electro-acupuncture/acupressure versus control (all types): SMD=-0.02 ( -0.17, 0.12) | |  |  |  |  |
| ***Quality of Life*** | | | | | | | | | | |
| Zeng, 2013 | Change in general QoL scores | | | 3 (121) | Acupuncture versus sham acupuncture: SMD=0.99 (-0.70, 2.68) | | Acupuncture showed no favorable effect in improving QoL when compared to sham acupuncture at 10-week follow-up. | | These three studies had low RoB. Considerable heterogeneity was found among the three studies (I^2^=92%, random effect model was used). | |
| Chen, 2013 | QLQ-C30 total score | | | 2 (85) | Acupuncture versus conventional care: SMD=0.47 (0.04, 0.09) | | Acupuncture can improve QoL for lung cancer patients as compared to conventional care. | | The SR authors did not provide details on RoB assessment for these two studies. | |
| Cheon, 2014 | Responder rate (% in Karnofsky score) | | | 1 (108) | No meta-analysis conducted | | Acupoints injection significantly improved QoL compared to conventional care (responder rate: 50% versus 25%, p<0.01). | | Evidence was reported from one single study (n=108). Details on RoB of this study were not provided by the SR authors. | |
| Lian, 2014 | QoL assessment scales and various indexes | | | 2 (159) | No meta-analysis conducted | | Both studies suggested that acupuncture plus conventional care can significantly improve quality of life in cancer patients compared to conventional care alone. | | These two trials were judged to have 2 or 3 scores out of 5 in the Jadad scale. However, no rationale was provided on how these scorings were rated. | |
| ***Other symptoms*** | | | | | | | | | | |
| Lu, 2007 | Leucopenia (change in WBC count | | | 7 (334) | Acupuncture +conventional care versus conventional care: WMD=1.22 (0.64, 1.81) | | Results from meta-analysis suggested that acupuncture plus conventional care was an effective option for chemotherapy-induced leukopenia as compared to conventional care alone. | | All the included studies were judged to be of high RoB under the Jadad scale. However, no rationale was provided on how these scorings were rated. | |
| Chao, 2009 | RILP | | | 1 (7) | No meta-analysis conducted | | The authors reported that WBC level were increased but no further details were provided. | | The result is reported from a small case series study. | |
| Chao, 2009 & Dos Santos, 2010 | BCRL | | | 1 (29) | No meta-analysis conducted | | In both SRs, acupuncture was found to be effective in treating BCRL. It is mentioned that the participants’ body circulation was enhanced, and sense of heaviness was reduced. Patients have also reported significant improvement on the range of movements, including shoulder flexion and abduction of the affected limbs. | | The result is reported from a small case series study | |
| Choi, 2012b | Response rates on reducing hiccup | | | 3 (162) | Acupuncture versus conventional care: RR=1.87 (1.26, 2.78) | | Results from meta-analysis suggested a favorable effect of acupuncture on response rate for patients’ hiccup as compared to conventional care. | | All the three studies had poor reporting quality on RoB related information, and were judged to have unclear RoB. | |
| Cheon, 2014 | Response rates on reducing hiccup | | | 2 (79) | No meta-analysis conducted | | Both trials reported a higher responder rate in the acupoints injection group as compared to conventional care. Result from one study reached statistically significance while the other did not. | | The two studies had unclear RoB for allocation concealment and high RoB for blinding. | |
| O'Sullivan, 2011 | Improvement on Xerostomia as measured by SFR | | | 3 (123) | No meta-analysis conducted | | All three trials reported significant improvement on SFR when compared to baseline (p<0.05), but no significant differences were seen between acupuncture and sham acupuncture group. | | All the three studies used objective outcome measurements to reduce detection bias. One trial has low RoB for allocation concealment while the other two have unclear RoB. | |
| Dos Santos, 2010 | Rating scale on dyspnea. | | | 1 (47) | No meta-analysis conducted | | In both groups, significantly improvement on dyspnea scores were observed immediately after treatment (p=0.003). Dyspnea scores were slightly higher in acupuncture group than sham group, however, no significant differences were seen. | | The trial was judged to have low RoB, but there is a lacking of blinding of personnel. | |

Keys: * Effects on dichotomous data were summarized with pooled risk ratio (RR), which measures the risk of experiencing certain outcome in the treatment group as compared to the control group across trials. Pooled standard mean difference (SMD) or weighted mean difference (WMD) were used for continuous outcomes. SMD was used if a variety of measurement methods were used for the same outcome, while WMD was used when outcome was measured with the same method.

BCRL, breast cancer-related lymphedema; CHM, Chinese herbal medicine; CI, confidence interval; CINV, chemotherapy induced nausea and vomiting; CRF, cancer related fatigue; CRP, cancer related pain; MFI, multidimensional fatigue inventory; QoL, quality of life; RCT, randomized controlled study; RILP, radiotherapy induced leukopenia; RoB, risk of bias; RR, risk ratio; SFR, salivary flow rates; SMD, standard mean difference; SR, systematic review; TENS, Transcutaneous electrical nerve stimulation; VAS, Visual analog scale for pain; WBC, white blood cell; WMD, weighted mean difference.
